# Mothers’ neural responses to infant faces are associated with activation of the maternal care system and observed intrusiveness with their own child

**DOI:** 10.3758/s13415-018-0592-6

**Published:** 2018-04-12

**Authors:** Joyce J. Endendijk, Hannah Spencer, Anneloes L. van Baar, Peter A. Bos

**Affiliations:** 1 Child and Adolescent Studies, Utrecht University, Heidelberglaan1, P.O. Box 80140, 3508 TC Utrecht, The Netherlands; 2 Department of Experimental Psychology, Utrecht University, Utrecht, The Netherlands

**Keywords:** Parenting quality, Event-related potentials, P1, P2, LPP, Mothers

## Abstract

Certain infant facial characteristics, referred to as *baby schema*, are thought to automatically trigger parenting behavior and affective orientation toward infants. Electroencephalography (EEG) is well suited to assessing the intuitive nature and temporal dynamics of parenting responses, due to its millisecond temporal resolution. Little is known, however, about the relations between neural processing of infant cues and actual parenting behavior in a naturalistic setting. In the present study we examined the event-related potentials (ERPs) of mothers (*N* = 33) watching infant faces of varying attractiveness, in relation to activation of the maternal care system and the mothers’ observed parenting behavior (sensitivity, nonintrusiveness) with their own child (2–6 years old). The results revealed that, irrespective of the cuteness of the infant face, mothers’ neural processing of infant faces involved both early P1 and P2 components (related to orienting/detecting processes) and late positive potentials (LPPs; related to more controlled cognitive evaluation/attentional engagement). Increased early detection and processing of infant faces (reflected by P1 and P2 activity) was related to increased activation of the parental care system. In later stages of face processing, increased attentional engagement with infant faces (as reflected by LPP activity) was associated with more intrusiveness of a mother with her own child during interaction. These findings suggest that individual variations in responses to infant stimuli are associated with individual differences in parental care system activation and parenting quality. Furthermore, the parental care system might be activated relatively automatically, but actual parenting and caregiving behavior requires more conscious control.

The importance of parental care for the optimal development of children is unequivocal and has been evident in decades of research (Ainsworth & Bell, 1970; Belsky & Jaffee, 2006; Bowlby, 1988; Groh, Roisman, van IJzendoorn, Bakermans-Kranenburg, & Fearon, 2012). Surprisingly, the body of research on the determinants of parental care and parenting quality is much smaller and mostly focused on extreme forms of nonoptimal parenting, such as child maltreatment (Belsky & Jaffee, 2006) and neglected intuitive/automatic processes (Papousek & Papousek, 2002). Knowledge of the determinants of the wide variation in parental care and parenting quality is essential for designing effective interventions that aim to enhance child development by enhancing parenting quality. A neuroscientific approach is a promising direction to take when examining the intuitive/automatic processes underlying parenting (Parke, 2017). A small but emerging body of research has successfully associated individual differences in the neural responses of parents to infant stimuli with variations in observed parenting behavior (Feldman, 2015). Rapid progress in the neuroscience of parenting is most likely to occur with research designs that build a bridge between neuroscientific measures and actual parenting behavior (Derks, Scheepers, & Ellemers, 2013; Feldman, 2015). Therefore, in the present study we used electroencephalography (EEG) to examine mothers’ neural responses to unfamiliar infant faces in relation to activation of the parental care system and to their parenting behaviors with their own child.

The parental care system and parenting behavior are two distinct, but related, constructs. The parental care system can be viewed as a motivational system: a coordinated set of affective and cognitive mechanisms, motivating parents, but also nonparents, to provide protection and nurturance for a child (Buckels et al., 2015; George & Solomon, 2008). Activation of the parental care system can be inferred from the presence of emotions, cognitions, and actions that facilitate protection and nurturance of children, including a positive attitude toward children (liking), willingness to take care of children and protect them from harm, and the tendency to experience tenderness across a variety of situations involving children (Buckels et al., 2015). Parenting behavior can be viewed as the result of activation of the parental care system (Bowlby, 1988). Two of the most important parenting behaviors in early childhood are parental sensitivity and nonintrusiveness (Ainsworth, Blehar, Waters, & Wall, 1978). *Sensitivity* refers to the adult’s ability to notice child signals, to interpret these signals correctly, and to respond to them promptly and appropriately (Ainsworth et al., 1978). *Nonintrusiveness* refers to the parent’s ability to refrain from behavior that is overdirecting, overstimulating, or interfering in the child’s activities (Biringen, 2008). Many studies have emphasized the importance of the activation of the parental care system as well as of high-quality parenting behaviors, such as sensitivity and nonintrusiveness, for optimal child development (e.g., Bakermans-Kranenburg, van IJzendoorn, & Juffer, 2003; Cabrera, Shannon, & Tamis-LeMonda, 2007; Hofer, Buckels, White, Beall, & Schaller, 2017).

The child itself has enormous power to evoke parenting. The caregiving system can be activated by cues that signal discomfort or distress in the child (e.g., crying; George & Solomon, 2008), but also by affectively rewarding infant cues (e.g., smiles, infant facial characteristics; Buckels, et al., 2015; Pryce, 1995). According to Lorenz (1943) certain infant facial characteristics (large forehead, big eyes, chubby cheeks, small nose and mouth), which he referred to as baby schema, automatically trigger the “Kindschenschema,” an innate releasing mechanism for parenting behavior and affective orientation toward infants (Hahn & Perrett, 2014; Langlois, Ritter, Casey, & Sawin, 1995). More specifically, the “Kindschenschema” can be considered as a biological mechanism, automatically generating caretaking and orienting responses to infants, with the evolutionary function of increasing survival chances of the infant (Glocker et al., 2009; Lorenz, 1943; Luo, Li, & Lee, 2011). This construct is thus highly similar to the definition of the parental care system.

The parental care system is, however, not activated with identical frequency and magnitude in all people, because of individual differences in biological and experiential factors (Buckels et al., 2015; George & Solomon, 2008). This might explain individual differences in actual parenting behavior. More specifically, individual variation in responsiveness to baby schema might be associated with individual differences in activation of the parental care system and parenting behavior. For example, people who respond strongly to infant facial stimuli might be better prepared biologically for parental caretaking. Indeed, women who find cuteness in infant faces highly rewarding have been found to report stronger maternal tendencies than women who find infant cuteness less rewarding, but there was no association between cuteness sensitivity and maternal tendencies (Hahn, DeBruine, & Jones, 2015). Also, increased attention/orienting to infant facial cues might facilitate the detection and interpretation of a child’s signals, which is an essential prerequisite for sensitive parenting behavior (Ainsworth & Bell, 1970). Parents’ sensitivity to subtle variations in infant facial characteristics might be a particularly important determinant of parenting, as parents need to be able to respond appropriately to subtle infant cues as well as to more obvious cues, like crying or smiling (Biringen, 2008).

A person’s sensitivity to baby schema has been examined in two ways: by comparing responses to infant versus adult faces, or by manipulating cuteness of infant faces (increasing or decreasing infantile features such as large forehead and eyes; Glocker et al., 2009). In the present study, the infant cuteness manipulation approach was employed. An advantage of this cuteness manipulation approach is that the same infant face is used to create both high- and low-cute versions. This controls for individual facial differences unrelated to baby schema, such as hairstyle, eye color, or facial symmetry, a problem that could confound responses to infant versus adult faces.

In addition, parents’ responsiveness to baby schema might not only influence parental care with baby’s and infants, but also set the stage for parenting with older children. Previous longitudinal research has demonstrated the relative consistency of parenting behaviors, such as sensitivity, from infancy to early childhood (Hallers-Haalboom et al., 2017). This indicates that parents who are highly sensitive with infants, possibly because of their high responsiveness to baby schema, are likely to be highly sensitive with older children, as well. Therefore, in the present study we examined mothers’ neural responsiveness to baby schema in relation to parenting behavior with infants as well as with older children.

Several studies have employed EEG and event-related potentials (ERPs) to examine neural responses to infant face stimuli (for reviews, see Maupin, Hayes, Mayes, & Rutherford, 2015; Young, Parsons, Stein, Vuust, Craske, & Kringelbach, 2017). However, only two of these studies have linked parents’ neural responses to infant faces to actual parenting behavior. One found no significant associations (Bick, Dozier, Bernard, Grasso, & Simons, 2013), whereas the other demonstrated that a larger difference in ERP responses (N170) to emotional versus neutral faces was related to higher maternal sensitivity (Bernard, Simons, & Dozier, 2015). Also, different experimental tasks were used, making comparisons between these studies difficult. The intuitive nature and temporal dynamics of parenting can be reflected in the millisecond temporal resolution of ERPs; therefore, more EEG studies on parenting are needed (Maupin et al., 2015). Furthermore, ERPs enable us to tease apart at what stage of the processing of infant cues individual differences in parenting emerge, and whether this happens at the level of perception, attentional processing, or cognitive evaluation/control (Young et al., 2017).

Regarding early ERP components, related to automatic perceptual/attentional processes, P1 activity to unfamiliar infant faces was found to be enhanced in women as compared to men, irrespective of infant facial expression (Proverbio, Brignone, Matarazzo, Del Zotto, & Zani, 2006). Furthermore, N170 activity was larger in response to unfamiliar infants showing negative affect than to those showing positive affect in men, women, fathers, and mothers (Peltola et al., 2014; Proverbio et al., 2006), although this effect was not consistently found (e.g., Malak, Crowley, Mayes, & Rutherford, 2015; Noll, Mayes, & Rutherford, 2012). Yet other studies have demonstrated enhanced early neural processing (N170, P2) of infant faces as compared to adult or older children’s faces (Hahn et al., 2016), particularly in women (Proverbio, Riva, Martin & Zani, 2010; Proverbio, Riva, Zani, & Martin, 2011). In sum, early components (e.g., P1, N170, P2) are enhanced toward infant facial cues and might be further affected by facial expressions.

Regarding later ERP components, which are related to cognitive evaluation and attentional engagement, P600/late positive potentials (LPPs) were found to be enhanced to a mother’s own infant’s face relative to unfamiliar infant faces (Bornstein, Arterberry, & Mash, 2013; Grasso, Moser, Dozier, & Simons, 2009). Furthermore, LPP activity has been found to be enhanced to infant as compared to adult or older children’s faces (Hahn et al., 2016), particularly in women (Proverbio et al., 2010; Proverbio et al., 2011). In sum, later processing stages (measured with the LPP component) were also found to be enhanced toward infant faces and were affected by infant familiarity.

Very few studies have specifically examined the effects of baby schema modulation on the neural processing of infant stimuli. Moreover, these studies focused on nonparents instead of parents. Hahn et al. (2016) showed that enhanced early (N170, P2) and later (LPP) processing of infant relative to adult faces was independent from the aesthetic quality (cuteness) of the faces, but that the cuteness of both infant and adult faces modulated early N170/P2 activity. Furthermore, Glocker et al. (2009) showed that manipulating the baby schema (i.e., cuteness) of infant faces modulated activation (fMRI) in neural regions associated with the processing of rewards (i.e., nucleus accumbens). This provides support for the idea that infant cuteness may modulate the activation of baby schema by influencing the processing and reward value of infant faces.

In the present study, we associated mothers’ neural responses to infant faces with activation of the parental care system and with parenting behaviors toward their own child. According to the premises of the “Kindschenschema” (Lorenz, 1943), we expected that increased neural processing of infant faces (P1, N170, P2, and LPP, based on Hahn et al., 2016) by mothers would be associated with increased activation of the parental care system and with higher-quality parenting behaviors with their own children. We also examined whether mothers’ neural responses to infant faces were modulated by infant cuteness.

## Method

### Participants

A total of 37 mothers with a child between 2 and 6 years old were recruited via the university website, parenting websites, and leaflets handed out in child-care centers. We aimed to include around 30 participants, in order to have enough power (.80) to detect a medium effect size (*f* = .25) with an alpha of .05 in a repeated measures analysis of variance (ANOVA) with three measurements (G*Power: Faul, Erdfelder, Buchner, & Lang, 2013). Four of the participants were excluded due to excessive noise and artifacts (>25% of trials) in their EEG recordings, resulting in a final sample of 33 mothers. See Table 1 for the demographic characteristics of the mothers and children. The Ethics Committee of the Faculty of Social and Behavioral Sciences of Utrecht University approved the study, which was performed in accordance with the latest version of the Declaration of Helsinki.

**Table 1:** Demographic characteristics of the sample

	*M*(*SD*)	Range
Mothers’ age	34.18 (4.57)	26–44
% highly educated^a^	80%	
% Dutch Caucasian ethnicity	94%	
Number of children		
1	39%	
2	49%	
3	12%	
Marital status		
Married/registered partnership	63%	
Cohabiting	28%	
Single-mother	9%	
Child age	3.15 (1.42)	2–6
% boys	54%	

^a^i.e., higher vocational or university level

### Procedure

First, behavioral observation of mother–child interactions took place. Mother and child were seated in a lab room with a table and chairs and no further distractions. Mothers were presented with a picture book and were told to look at all the pictures and to talk to their child about what they saw in the pictures, for a maximum of 10 min. After the instruction, the experimenter left the room. The interaction was filmed and coded afterward.

For the EEG assessment, each mother was taken to another lab room by a second experimenter. The child stayed with the first experimenter in the behavioral lab. During EEG recording, participants were seated in a soundproof, normally lighted room without windows. They were instructed to minimize eye or body movements during the recording period. After the EEG tasks, each mother completed several online questionnaires on her child’s behavior and her own parenting practices. Only the parental care questionnaire (see below) was relevant for the research questions in the present study. The other questionnaires assessed child behavior problems, gender-typical behavior of the child, and mothers’ gender stereotypes. The mothers and children, respectively, received a financial compensation (€15) and a small present for their participation.

### Measures

#### Observed parenting behaviors

Two dimensions of mothers’ parenting behaviors were assessed with the Emotional Availability Scales, fourth edition (EAS; Biringen, 2008): sensitivity and nonintrusiveness. *Sensitivity* refers to the parent’s ability to be warm and appropriately responsive to the child. *Nonintrusiveness* refers to the parent’s ability to give the child space to explore and to refrain from intrusions on the child’s activities. Each dimension is divided into seven subscales; the first two subscales are coded on a 7-point Likert scale, and the other subscales are coded using a 3-point Likert scale (potential score range 7–29). Subscale 7 of the nonintrusiveness dimension (the adult is made to “feel” or “seem” intrusive) was excluded because it refers to child behavior rather than parental behavior (leading to a potential score range of 7–26). For more information about this measure, see Hallers-Haalboom et al. (2014). The first author, who is an experienced coder of parent–child interactions, trained one undergraduate student to code the videos for sensitivity and nonintrusiveness. Coder reliability was determined on a previously developed reliability set (Hallers-Haalboom et al., 2014, *n* = 30). Coder reliability was adequate: The intraclass correlation coefficients (the absolute agreement between the student scores and consensus scores) were .85 for sensitivity and .82 for nonintrusiveness. During the coding process, the first ten videotapes were coded twice by the first author and the student coder, and disagreements were discussed until consensus had been achieved.

#### Self-reported activation of parental care system

The validated Parental Care and Tenderness Questionnaire (PCAT: Buckels et al., 2015) was used to assess individual differences in the activation of the parental care system. The questionnaire was translated into Dutch by author H.S. and one independent bilingual Dutch and English speaker using forward- and back-translation. Discrepancies were resolved after discussion with author P.A.B. Because the parental care system can be activated in both parents and nonparents, all items referred to children generally (i.e., no items referred to one’s own offspring). This ensures applicability to both parents and nonparents. First, mothers indicated their agreement with 15 statements covering a positive attitude toward infants (liking: e.g., “I don’t like to be around babies (reverse coded).”) and a willingness to care for infants (caring: e.g., “When I see infants, I want to hold them”) and protect them (protection: e.g., “I would use any measures necessary to protect a child, even if I had to hurt others.”), on a 5-point scale (1 = *strongly disagree* to 5 = *strongly agree*). Second, mothers indicated the amount of tenderness they would feel in ten specific situations involving infants (positive situations [e.g., “A newborn baby curls its hand around your finger.”] and negative situations [e.g., “You see that a baby is sick.”]) on a 5-point scale (1 = *no tenderness at all* to 5 = *a lot of tenderness*). A factor analysis revealed one dominant factor (eigenvalue = 7.13, variance explained = 28.53%). Therefore, the scores were averaged in one composite score (Cronbach’s alpha = .86), with higher scores indicating greater activation of the parental care system.

#### Infant cuteness task

**Fig. 1:**
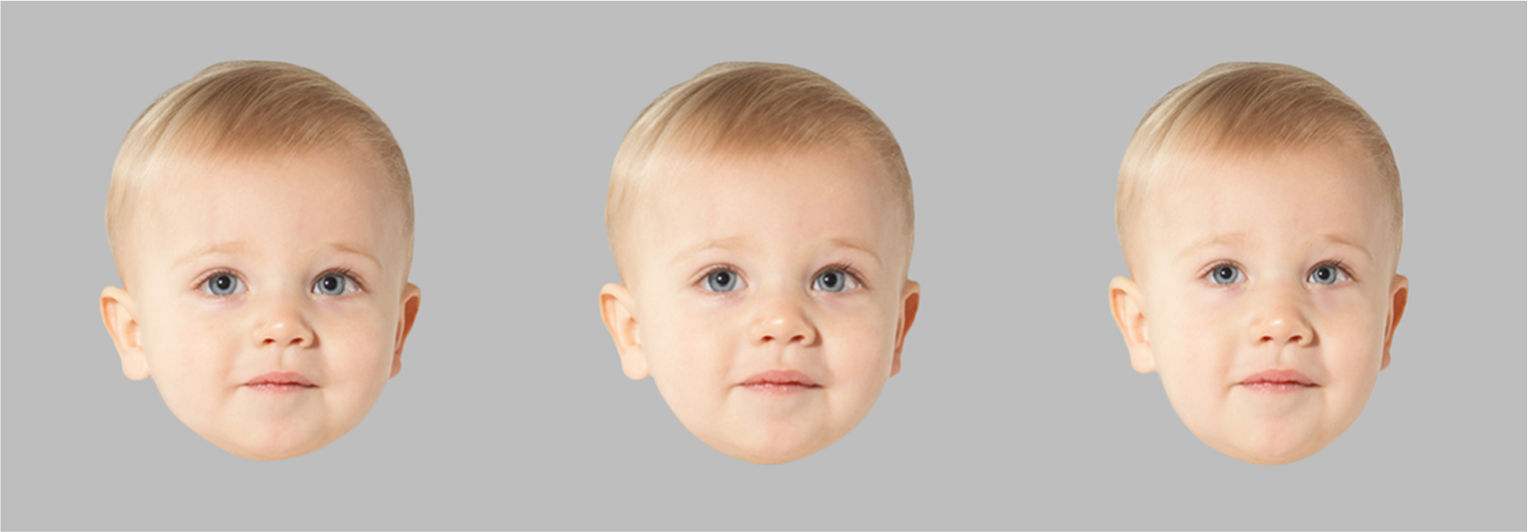
Examples of high-cute (left), normal (middle), and low-cute (right) infant stimuli. Photos taken from “Baby Schema in Human and Animal Faces Induces Cuteness Perception and Gaze Allocation in Children,” by M. Borgi, I. Cogliati-Dezza, V. Brelsford, K. Meints, & F. Cirulli, Borgi et al., 2014, *Frontiers in Psychology*, *5*, 411, Fig. 2. Copyright 2014 Frontiers Media S.A. Adapted by permission.

To assess neural responses to the infant stimuli, mothers completed a task adapted from Glocker et al. (2009) in which they had to watch infant faces of varying levels of cuteness. High-cute and low-cute prototypes of the same infant faces have previously been developed (Borgi, Cogliati-Dezza, Brelsford, Meints, & Cirulli, 2014; Glocker et al., 2009). On the basis of Borgi et al.’s and Glocker et al.’s work, each infant face (nine in total) was transformed in cuteness to create a low-cute version and a high-cute version of the face (see Fig. 1). The techniques and procedures used to create the baby schema stimuli are reported in detail elsewhere (Borgi et al., 2014; Glocker et al., 2009). To summarize, baby schema features were captured by six facial parameters: absolute face width (fw) in pixels with head length fixed and five proportion indices—forehead length/face length (fol/fal), eye width/face width (ew/fw), nose length/head length (nl/hl), nose width/face width (nw/fw), and mouth width/face width (mw/fw). The baby schema content in each image was manipulated using the range of baby schema values (mean and *SD*) from a sample of unmanipulated images as a guide for the manipulation procedure. Using Photoshop, these facial parameters were manipulated in nine infants, to produce high-cute (round face, high forehead, big eyes, small nose and mouth: fw, fol/fal, ew/fw > mean, nl/hl, nw/fw, mw/fw < mean) and low-cute (narrow face, low forehead, small eyes, big nose and mouth: fw, fol/fal, ew/fw < mean, nl/hl, nw/fw, mw/fw > mean) versions of each infant face. The Photoshop resize tool was used to enlarge or reduce forehead length, nose length, face width, eye width, nose width, and mouth width; clone stamp and healing brush tools were used to adjust sections of the picture that appeared unnaturally stretched. To maintain normal facial appearance, the manipulation for each facial parameter was restricted to a *z*-score range of ±2 *SD*s. Only those parameters that needed an adjustment in order to obtain a high- or low-cute facial characteristic were manipulated.

In the EEG task, the 27 high-cute, low-cute, and normal infant faces were each presented once on a 19-in. Dell monitor in a semi-random way, such that each identity was presented once per block (total number of trials = 27). Each trial began with the presentation of a black fixation cross at the center of a gray background (rgb: 190, 190, 190) for 1,000 ms. An infant face was then displayed in the center of the screen for 2,000 ms. After infant face offset, participants were prompted to rate the cuteness of the infant face on a 10-point scale (0 = *not at all cute*, 9 = *very cute*), followed by a rating of their willingness to take care of the infant on a 10-point scale (0 = *not at all willing to take care of this infant*, 9 = *very willing to take care of this infant*). Responses were provided by using the buttons 0–9 on a keyboard. Response time was unlimited, and the trial ended when both responses had been made. Trials were separated by an interstimulus interval of 1,000 ms. Stimulus presentation, timing, and the measurement of behavioral response time and accuracy were controlled by the E-Prime (version 2.0) software (Schneider, Eschman, & Zuccolotto, 2002).

Following Hahn et al. (2015), we calculated a *cuteness sensitivity score* by subtracting the mean ratings that mothers gave to the low-cuteness versions of infant faces from the ratings they gave to the high-cute versions. Higher scores indicated that cuteness had a greater effect on ratings.

#### EEG assessment

EEG was continuously recorded from 32 scalp sites, using BioSemi ActiveTwo Ag–AgCl pin electrodes and hardware (Biosemi, Amsterdam, The Netherlands). The electrodes were placed according to the 10–20 electrode system (Klem, Lüders, Jasper, & Elger, 1999), using a nylon electrode cap. The EEG signals were amplified with a bandpass of DC 400 Hz by BioSemi ActiveTwo amplifiers, sampled at 2048 Hz. Vertical and horizontal bipolar electrooculographic activity (EOG) was recorded in order to monitor eye movements using sintered Ag–AgCl electrodes placed above and below the right orbit and near the outer canthus of each eye.

Offline processing of EEG activity was performed with Brain Vision Analyzer (version 2.1). First, the data were downsampled to 256 samples per second, followed by bandpass filtering between 0.1 and 30 Hz. The data were re-referenced to the average activity of all electrodes. The Gratton et al. method with raw average subtraction (Gratton, Coles, & Donchin, 1983) was used to correct for eye movements and blinks. Epochs time-locked to the onset of the infant face stimuli were extracted from the cleaned data using a time window of – 200 to 1,000 ms. A period of 200 ms before stimulus onset was used for baseline correction. Artifacts were detected and rejected semi-automatically. Trials with the following characteristics were manually inspected: with a lowest activity below 0.5 *μ*V, a peak-to-peak voltage greater than 150 *μ*V, a maximum allowed amplitude of ±100 *μ*V, or a maximum allowed voltage step that exceeded 50 *μ*V, all within a 200-ms moving window. These trials were deleted when an artifact was visible in multiple electrodes or at one of the electrode sites of interest (isolated artifacts on electrodes outside the region of interest were present on average in <1% of all trials). A channel was marked as “bad” if noise levels were significantly larger than on other channels (>25% of trials rejected due to artifacts exclusively present on “bad” channels). Subsequently, “bad” channels were removed from all preprocessing steps and further analyses. For three participants, one or two channels were removed from the datasets. On average, 5% of trials (range: 0–22%) were rejected due to artifacts. This means that per person, on average 8.57 trials were included in the analyses per condition (no differences in included trials between conditions, *p* = .24). The remaining trials were averaged in a grand average waveform (average number of trials per person = 25.70), but also separately for each condition (high-cute, low-cute, normal).

#### ERPs

The time windows and electrodes were chosen on the basis of visual inspection of the grand average waveforms (i.e., electrodes with the strongest peak activity) and topographical maps (see Fig. 3 below). We focused on electrodes with the largest amplitudes for each ERP component. All ERP components were quantified as the average activity in a discrete time window following stimulus presentation in the grand average waveform. Time windows based on the grand average waveforms were verified in each individual subject and expanded when necessary to capture individual variability. Specifically, the P1 was quantified poststimulus from 100 to 150 ms (measured over P7, P8, PO3, PO4, O2, and O1), the N170 from 150 to 230 ms (measured over P7 and P8), the P2 from 250 to 350 ms (measured over PO3, PO4, O1, and O2), and the LPP from 300 to 700 ms (measured over Pz). The average (mean) activity in these time windows was exported to SPSS. These time windows and electrode sites are similar to those from previous ERP studies using face stimuli (Hahn et al., 2016; Werheid, Schacht, & Sommer, 2007).

### Analyses

#### Descriptive analyses

As a manipulation check, the cuteness/caretaking ratings of infant faces were submitted to a repeated measures ANOVA with condition (high-cute, normal, low-cute) as a within-subjects factor. Correlations were computed between mothers’ behavioral assessments of parenting (sensitivity, nonintrusiveness, activation of parental care system) and cuteness/caretaking ratings.

#### Hypothesis testing

The averaged ERP activity across electrodes for the P1, N170, P2, and LPP components was submitted to a repeated measures ANOVA with condition (high-cute, normal, low-cute) as a within-subjects factor (analyses including hemisphere or electrode did not yield significant interactions with condition). Appropriate corrections for sphericity were made when necessary (Greenhouse–Geisser correction when *ε* < .75; Huyn–Feldt correction when *ε* > .75). When significant differences in ERPs were found between the high-cute and low-cute conditions, associations between the behavioral parenting data and ERPs were examined by including the behavioral variable as a covariate in the repeated measures ANOVAs. When no significant differences in ERPs were found between conditions, the ERPs were averaged across conditions and correlated with the self-reported and observed parenting data.

## Results

### Descriptive analyses

**Fig. 2:**
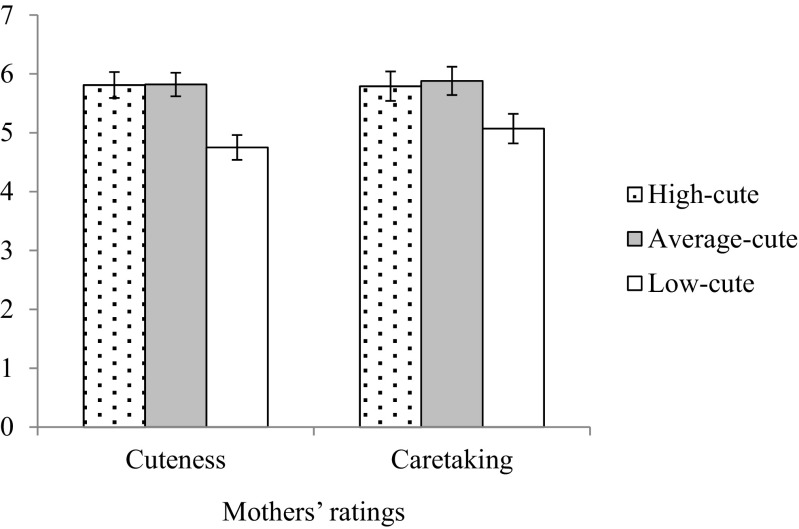
Mothers’ ratings of high-cute, normal, and low-cute infant faces. Ratings represent cuteness and willingness to take care of the infant in the picture. Error bars represent standard errors of the means.

Figure 2 shows average cuteness and caretaking ratings for infant faces in the high-cute, normal, and low-cute conditions. Mothers rated high-cute and normal infants as significantly cuter than the low-cute infants [condition effect: *F*(1, 31) = 46.77, *p* < .001, *η*_p_^2^ = .75; contrast high-cute/low-cute: *t*(32) = – 9.38, *p* < .001; contrast normal/low-cute: *t*(32) = – 9.45, *p* < .001]. There were no differences in cuteness ratings between the high-cute and normal infants, *t*(32) = 0.21, *p* = .84. Similarly, mothers indicated that they were significantly more willing to take care of the high-cute and normal infants than of the low-cute infants [condition effect: *F*(1, 31) = 26.07, *p* < .001, *η*_p_^2^ = .63; contrast high-cute/low-cute: *t*(32) = – 7.10, *p* < .001; contrast normal/low-cute: *t*(32) = – 6.66, *p* < .001]. No differences were found in willingness to take care of the high-cute and normal infants, *t*(32) = 1.14, *p* = .26. Both effects of condition on mothers’ ratings were large.

Table 2 shows descriptive statistics and the correlations between mothers’ ratings of infant faces, parental care, and observed maternal sensitivity and nonintrusiveness with their own child. Cuteness and caretaking ratings were strongly associated across the high-cute, low-cute, and normal conditions. Observed maternal sensitivity was associated with nonintrusiveness. Self-reported activation of the parental care system was not associated with sensitivity, nonintrusiveness, or the cuteness and caretaking ratings. The cuteness and caretaking ratings were also not associated with either sensitivity or nonintrusiveness. Cuteness sensitivity was not associated with any of the parenting variables.

**Table 2:** Descriptive statistics and correlations for the behavioral data

	1. Cuteness High	2. Cuteness Normal	3. Cuteness Low	4. Cuteness Detection	5. Caretaking High	6. Caretaking Normal	7. Caretaking Low	8. Parental Care System Activation	9. Sensitivity	*M*(*SD*)
1. Cuteness high										5.81 (1.27)
2. Cuteness normal	.96^**^									5.82 (1.17)
3. Cuteness low	.86^**^	.85^**^								4.75 (1.21)
4. Cuteness sensitivity	.35^*^	.29	– .18							1.06 (0.65)
5. Caretaking high	.81^**^	.75^**^	.66^**^	.35^*^						5.79 (1.46)
6. Caretaking normal	.74^**^	.76^**^	.59^**^	.35^*^	.95^**^					5.88 (1.38)
7. Caretaking low	.70^**^	.66^**^	.71^**^	.04	.92^**^	.88^**^				5.07 (1.45)
8. Parental care system activation	.29	.26	.25	.11	.31	.28	.27			3.84 (0.46)
9. Sensitivity	– .08	– .08	– .19	.19	– .18	– .15	– .24	– .21		24.58 (3.09)
10. Nonintrusiveness	.10	.05	.09	.02	– .10	– .19	– .07	– .03	.51^**^	21.73 (2.41)

Cuteness and caretaking ratings were assessed in the EEG infant cuteness task. Parental care system activation was assessed with a self-report questionnaire. Sensitivity and nonintrusiveness were observed when mother and child were reading a book together.

^*^p < .05, ^**^p < .01.

None of the background variables were significantly related to the parenting variables (all *p*s > .12).

### EEG data

**Fig. 3:**
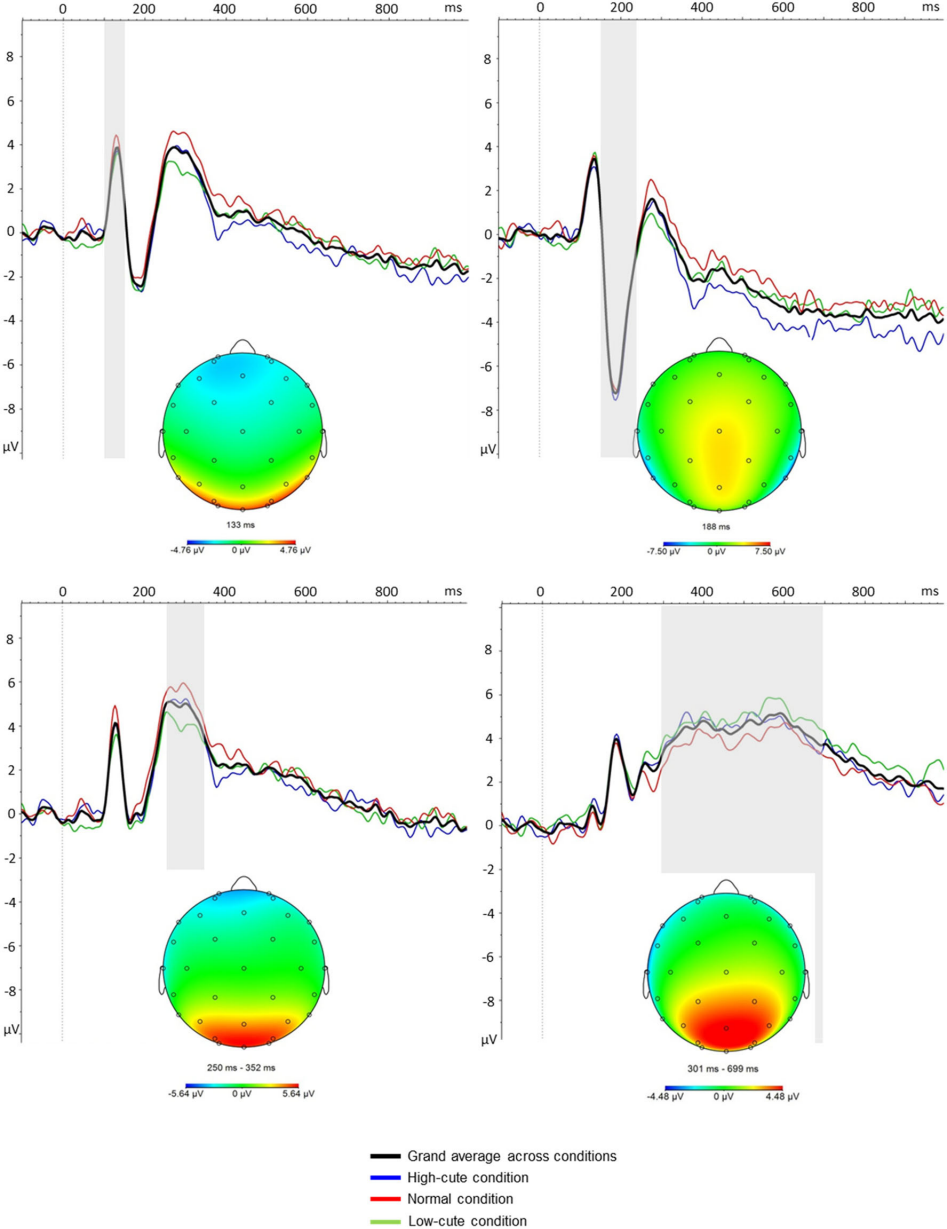
Grand average ERPs across conditions and separated for the high-cute, normal, and low-cute infant conditions. Gray areas in the panels represent the ERP time windows of interest: (top row left panel) P1 (measured over P7, P8, PO3, PO4, O1, and O2), (top row right panel) N170 (measured over P7 and P8); (bottom row left panel) P2 (measured over PO3, PO4, O1, and O2), (bottom row righ panel) and LPP (measured over Pz).

See Fig. 3 for grand average EEG waveforms across the different cuteness conditions. No significant differences were found between conditions in P1 activity [*F*(2, 64) = 0.81, *p* = .45, *η*_p_^2^ = .03], N170 activity [*F*(2, 64) = 0.24, *p* = .75, *η*_p_^2^ = .01], or LPP activity [*F*(2, 64) = 1.26, *p* = .29, *η*_p_^2^ = .04]. There was a difference between conditions in P2 activity [*F*(2, 64) = 4.99, *p* = .01, *η*_p_^2^ = .14]. However, follow-up *t* tests showed that only the difference between the low-cute and normal condition was significant [*t*(32) = 3.13, *p* = .01, Bonferroni corrected], with higher P2 activity in the normal condition. We observed no differences in P2 activity between the high-cute and low-cute conditions [*t*(32) = 1.98, *p* = .17, Bonferroni corrected] or between the high-cute and normal conditions [*t*(32) = – 1.28, *p* = .63, Bonferroni corrected]. Because no meaningful differences were found between the high- and low-cute conditions, and to reduce the amount of noise, the ERPs were averaged across conditions.

### Associations between parenting data and the EEG data

#### P1

**Fig. 4:**
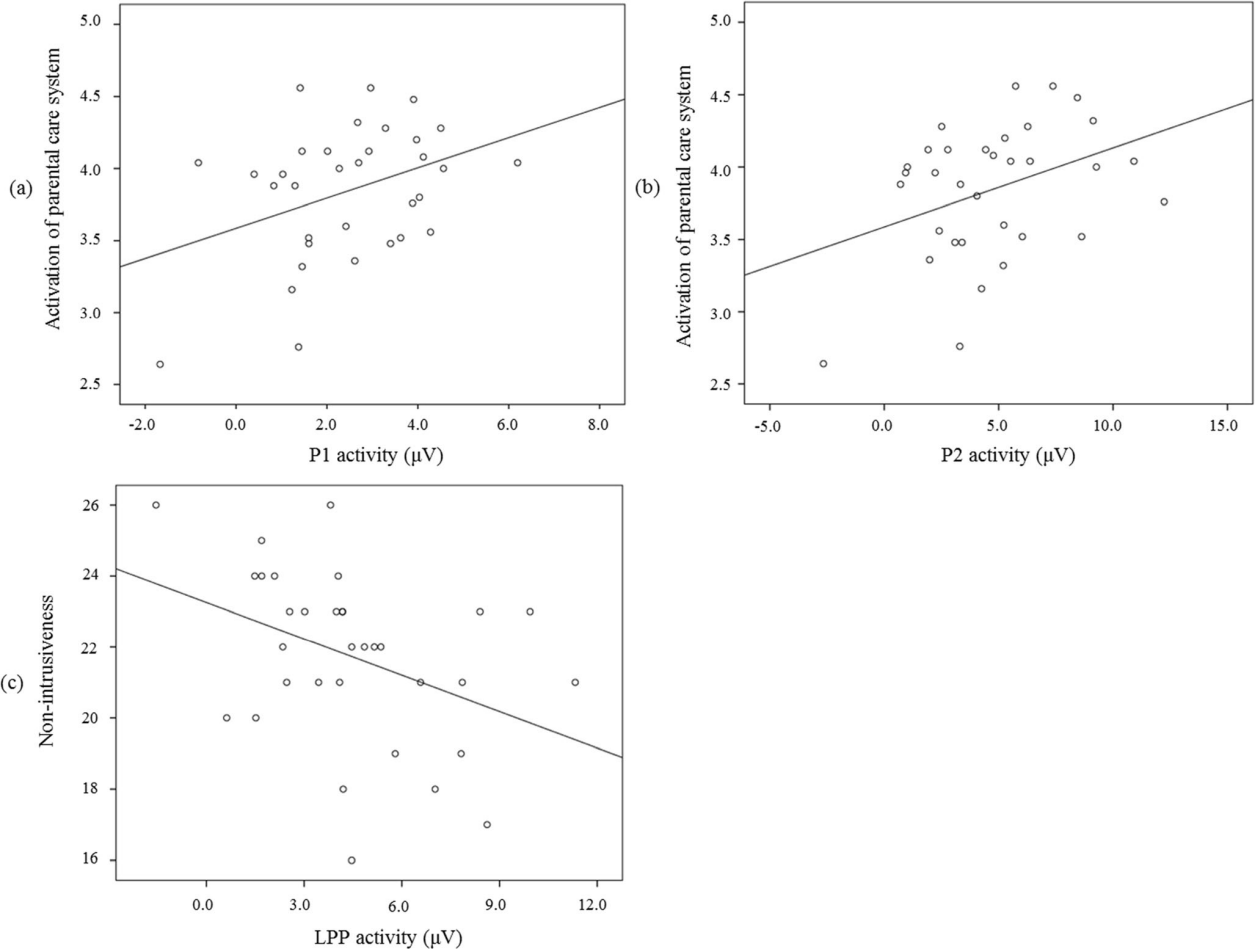
Associations between ERPs and parenting behavior for the (a) P1, (b) P2, and (c) LPP.

Increased P1 activity to infant faces was associated with stronger self-reported activation of the parental care system (*r* = .37, *p* = .03; see Fig. 4a). P1 activity to infant faces was not associated with observed parenting quality (all *r*s < .12, *p*s > .51).

#### N170

No significant associations were found between N170 activity to infant faces and the mothers’ behavioral data (all *r*s < – .25, *p*s > .17).

#### P2

Increased P2 activity to infant faces was associated with stronger self-reported activation of the parental care system (*r* = .37, *p* = .03; see Fig. 4b). No other significant associations were found between P2 activity to infant faces and the mothers’ behavioral data (all *r*s < – .09, *p*s > .63).

#### LPP

For the LPP, correlations were computed for the entire period from 300 to 700 ms. Increased LPP activity to infant faces was associated with decreased nonintrusiveness with a mother’s own child (i.e., increased intrusiveness) (*r* = – .40, *p* = .02; see Fig. 4c). LPP activity was not associated with either observed sensitivity or self-reported activation of the parental care system (all *r*s < .19, *p*s > .29).

## Discussion

In this EEG study, we innovatively examined mothers’ neural responses to infant faces of varying cuteness and linked these neural responses to actual parenting behavior. First, we found several associations between mothers’ neural responses to infant faces (high-cute, normal, and low-cute grouped together) and self-reported activation of the maternal care system and observed parenting quality. Increased P1 and P2 activity, which has been suggested to reflect early detection and processing of infant faces, was found to be related to mothers’ higher self-reported activation of the parental care system. Furthermore, enhanced LPP activity in later stages of face processing, which has been suggested to reflect increased attentional engagement with infant faces, was found to be associated with increased intrusiveness with a mother’s own child.

These findings are in line with ideas originating from the “Kindschenschema” (Lorenz, 1943). It appears that individual variation in neural responsiveness to baby schema in infant stimuli is associated with individual differences in activation of the parental care system and in the quality of parenting behaviors. Interestingly, mothers’ perceived cuteness of infants or willingness to take care of infants was not predictive of activation of the parental care system or of the quality of parenting behavior. Thus, variations in fast, automatic responsiveness to infant cues might be more important determinants of parenting than more conscious appraisal of infants and motivation for caretaking. Similarly, mothers’ self-reported sensitivity to infant cuteness was also unrelated to all of the parenting variables we assessed. This finding is in line with a previous study showing that not the ability to detect differences in infant cuteness, but rather the reward value of infant cuteness, is related to maternal tendencies (Hahn et al., 2015).

However, no support was found for the idea that increased attention to infant faces might facilitate the detection and interpretation of a child’s signals, which is an essential prerequisite for sensitive parenting (Ainsworth & Bell, 1970). In fact, mothers’ enhanced LPP components were associated with intrusive parenting behaviors that violated the child’s autonomy. These mothers might also pay excessive attention to their own child (i.e., monitoring), which is associated with overinvolved parenting behaviors (Ellis, Templin, Naar-King, & Frey, 2008). Moreover, mothers’ increased LPP amplitudes to neutral infant faces have also been found to be related to mothers’ anxiety symptoms (Malak et al., 2015). Maternal anxiety is a strong predictor of intrusive parenting behaviors that violate the child’s autonomy (van der Bruggen, Stams, & Bögels, 2008).

The fact that we did not find associations between mothers’ neural processing of infant faces and their sensitivity with her own child might have something to do with the setting in which we observed sensitivity. Variation in parental sensitivity is best captured in situations in which the infant shows distress (McElwain & Booth-LaForce, 2006), which was not the case in the current book-reading setting. Furthermore, the infant faces used in the EEG paradigm displayed neutral affect and no distress. Previous EEG studies have particularly demonstrated enhanced neural responses toward infant faces showing negative affect (Doi & Shinohara, 2012; Peltola et al., 2014; Proverbio et al., 2006). Future studies should examine whether neural processing of distressed infant faces is predictive of maternal sensitive responding to distress in their own infants. An alternative explanation for some of the non-significant associations between neural processing of infant faces and the parenting variables is a lack of power of this study. With a larger sample size (*N* > 100), we would have had more power to show the significance of smaller correlations, but the medium effect size that we needed in relation to our sample size also points out the stronger and probably clinically more important associations.

It is interesting to note that early neural processing of infant faces (as reflected by P1 and P2 activity) was specifically associated with self-reported activation of the parental care system. However, later, more controlled, processing of infant faces (as reflected by LPP) was specifically associated with actual parenting behaviors with mother’s own child. This suggest that the parental care system might be activated relatively automatic, but that actual parenting and caregiving behavior require more conscious control. Previous EEG studies demonstrated that both early automatic perceptual processes (orienting/detecting) and later more controlled cognitive evaluation and attentional engagement with infant faces are implicated in infant face processing (Bornstein et al., 2013; Doi & Shinohara, 2012; Grasso et al., 2009; Peltola et al., 2014; Proverbio et al., 2006, Proverbio et al., 2011). It was not yet known so far, how or why early and late processing would be relevant for parental caregiving.

Another finding that is important to highlight is the lack of association between activation of the parental care system and the quality of mothers’ parenting behavior with their own children. This could indicate that activation of the parental care system by infant cues does not necessarily lead to high quality parenting behavior toward children. Actual parenting behavior with one’s children is likely to depend on many more factors, such as parents’ evaluation of the child’s signals, evaluation of the context, and past experiences both as a child and a parent (George & Solomon, 2008). It is also possible that the relative safe and predictable book-reading setting in which we observed mother–child interaction might not have activated the parental care system that much, because the child did not need protecting or nurturing.

Furthermore, we found no evidence that infant cuteness modulated mothers’ neural responses to infants. This might be surprising considering the strong effects infant cuteness had on mothers’ ratings of infant cuteness and willingness to take care of the infants in the pictures. Moreover, attractiveness of faces in general has been found to modulate (enhance/reduce) early and later neural processing of faces (Chen et al., 2012; Hahn et al., 2016; Zhang & Deng, 2012). In addition, one previous study demonstrated that high-cute infant faces increased activation (fMRI) in neural regions associated with the processing of rewards (Glocker et al., 2009). However, in this fMRI study the manipulation of baby schema (i.e., cuteness) primarily elicited a striatal brain response, and such subcortical brain activity might have been difficult to detect with EEG (Cohen, Cavanagh, & Slagter, 2011). It is not likely that the cuteness manipulation was too subtle to elicit differential neural responses, because we used stimuli similar to those of Glocker et al. Also, mothers could clearly detect differences in cuteness between the high- and low-cute infants. Another possibility is that the low number of trials in this study reduced its power to detect differential responses. This substantiates our choice to group trials together for the analyses examining associations between neural responses to infant faces and mothers’ parenting behavior. However, the low number of trials may still have affected our results, in particular the association between LPP and maternal nonintrusiveness, since the reliability of sustained waveforms is reduced by a limited number of trials.

A more substantive explanation for our findings is related to the fact that Glocker et al. (2009) examined nulliparous young women. The present study is the first to examine effects of baby schema modulation (cuteness) in mothers. It is possible that high-cute infants are more rewarding to women without children, but that for mothers, infant faces might be rewarding regardless of cuteness. From an evolutionary viewpoint, it makes sense, for the survival of the infant, that mothers are equally responsive to all infants (cute or not cute) regardless of variation in baby schema between infants. All infants to a certain extent possess facial characteristics that make up the baby schema. It appears that even though mothers can objectively and consciously detect differences in infant cuteness, they might be equally responsive to all infants at a more automatic/unconscious level (e.g., no difference in P1, N170, P2 activity).

Our null findings for the infant cuteness modulation on mothers’ N170 response to infant faces fit the claim that this ERP component predominantly reflects the structural encoding of face stimuli (Eimer & Holmes, 2007) and might be relatively unaffected by infant cuteness. However, the literature is inconsistent about whether variations in infant facial characteristics, such as emotional expressions, modulate N170 activity, with some studies finding an effect of infant face modulation (Peltola et al., 2014; Proverbio et al., 2006) and others not (Malak et al., 2015; Noll et al., 2012). Relatedly, Bernard et al. (2015) did find an association N170 activity to emotional infant faces and maternal sensitivity, whereas we did not find associations between N170 activity to infant faces and parenting. It is possible that infant emotions elicit stronger neural responses than neutral faces, which might make individual differences in sensitivity to infant cues better detectable. Thus, more studies are needed to help clarify the conditions under which enhanced sensitivity to infant facial cues has the greatest impact on parenting.

The results need to be interpreted while considering the limitations of this study. First, this study had a correlational design. Therefore, no firm conclusions could be drawn about whether neural processing of infant faces predicts parental care system activation and the parenting quality of mothers, or the other way around. Parenting intervention studies could provide clarity in this regard (Bernard et al., 2015), especially when parents’ neural responses to infant stimuli and parenting quality are both measured at multiple time points (e.g., pre-intervention, immediately post, follow-up). Such a design makes it possible to examine whether changes in ERP responses mediate pathways toward improved parenting, or the other way around.

Second, we used face stimuli of unfamiliar infants, even though own-infant faces have been found to elicit larger neural responses than other-infant faces (Bornstein et al., 2013; Doi & Shinohara, 2012; Grasso et al., 2009). However, sensible associations with mothers’ caregiving were found regardless of the use of unfamiliar infant faces.

Third, we focused specifically on mothers, but it should be realized that the processes involved might be different for fathers. Neural processing of infant stimuli might be reduced in fathers, because fathers appear to score lower on aspects of parenting quality such as sensitivity and nonintrusiveness (Hallers-Haalboom et al., 2014). This hypothesis should be tested empirically.

Fourth, we set out to examine mothers’ neural responsiveness to variations in baby schema in relation to parenting. However, no differences in neural processing were found of high-cute and low-cute infant faces. Therefore, we had to examine parenting in relation to neural processing of all infant faces (high-cute, low-cute, and normal grouped together). With this design we cannot know for sure whether the brain responses found are specific for processing of infant faces or more reflective of face processing in general. To prevent this problem, future studies could use a design in which both infant and adults faces are manipulated for cuteness (see Hahn et al., 2016).

Finally, we examined mothers with children in a wide age range, including infants, preschoolers, and school-aged children, which may have affected our results. Maternal interaction quality appears to increase between the ages of 2 and 6 (Hallers-Haalboom et al., 2014). However, in the present study, child age was unrelated to parenting quality, and controlling for child age did not change our findings. Future studies could examine whether neural responses to infant cues are more important determinants of parenting behavior in the infancy period than of parenting behavior toward older children. It is also important for future studies to examine whether parenting interventions can produce long-term change in parents’ intrusiveness and underlying neural processing of infant cues. Intrusive parenting behavior has been found to be associated with nonoptimal outcomes during early childhood, such as externalizing behaviors and lower academic achievement (e.g., Cabrera et al., 2007; Ispa et al., 2004).

To conclude, this study showed that mothers’ neural processing of infant faces involves both early orienting/detecting processes (as reflected by P1, N170, and P2 activity) and later more controlled cognitive evaluation and attentional engagement (as reflected by LPP). Furthermore, infant face processing appeared to be independent from infant cuteness. Individual variation in early and later stages of face processing was associated, respectively, with individual differences in activation of the parental care system and intrusive parenting behavior. Therefore, this study, combining neuroscientific measures with maternal responses, showed a clear differentiation in the relations between the underlying physiological processes and both maternal self-reports on the activation of the parental care system and maternal behavioral responses during interactions with their children.

### Author note

The work in this article was supported by a grant from the Netherlands Society of Scientific Research to PAB (451-14-015). Other than this, the research did not receive any specific grant from funding agencies in the public, commercial, or not-for-profit sectors. The authors report no conflicts of interest. We thank the mothers and children for participating in this study and Myrthe Mensink and Henny Tichelaar for assistance with recruiting participants and data collection, as well as Marta Borgi for kindly sharing her stimuli.

## References

[CR1] Ainsworth MDS, Bell SM (1970). Attachment, exploration, and separation: Illustrated by the behavior of one-year-olds in a strange situation. Child Development.

[CR2] Ainsworth MDS, Blehar MC, Waters E, Wall S (1978). *Patterns of attachment: A psychological study of the Strange Situation*.

[CR3] Bakermans-Kranenburg MJ, van IJzendoorn MH, Juffer F (2003). Less is more: Meta-analyses of sensitivity and attachment interventions in early childhood. Psychological Bulletin.

[CR4] Belsky J, Jaffee SR, Ciccetti D, Cohen DJ (2006). The multiple determinants of parenting. *Developmental psychopathology: Risk, disorder, and adaptation*.

[CR5] Bernard K, Simons R, Dozier M (2015). Effects of an attachment-based intervention on child protective services—Referred mothers’ event-related potentials to children’s emotions. Child Development.

[CR6] Bick J, Dozier M, Bernard K, Grasso D, Simons R (2013). Foster mother–infant bonding: Associations between foster mothers’ oxytocin production, electrophysiological brain activity, feelings of commitment, and caregiving quality. Child Development.

[CR7] Biringen, Z. (2008). *The Emotional Availability (EA) Scales* (4th ed., Infancy/Early Childhood Version, child age: 0–5 years). Retrieved from www.emotionalavailability.com

[CR8] Borgi M, Cogliati-Dezza I, Brelsford V, Meints K, Cirulli F (2014). Baby schema in human and animal faces induces cuteness perception and gaze allocation in children. Frontiers in Psychology.

[CR9] Bornstein MH, Arterberry ME, Mash C (2013). Differentiated brain activity in response to faces of “own” versus “unfamiliar” babies in primipara mothers: An electrophysiological study. Developmental Neuropsychology.

[CR10] Bowlby J (1988). *A secure base: Parent–child attachment and healthy human development*.

[CR11] Buckels EE, Beall AT, Hofer MK, Lin EY, Zhou Z, Schaller M (2015). Individual differences in activation of the parental care motivational system: Assessment, prediction, and implications. Journal of Personality and Social Psychology.

[CR12] Cabrera NJ, Shannon JD, Tamis-LeMonda C (2007). Fathers’ influence on their children’s cognitive and emotional development: From toddlers to pre-K. Applied Development Science.

[CR13] Chen J, Zhong J, Zhang Y, Li P, Zhang A, Tan Q, Li H (2012). Electrophysiological correlates of processing facial attractiveness and its influence on cooperative behavior. Neuroscience Letters.

[CR14] Cohen MX, Cavanagh JF, Slagter HA (2011). Event-related potential activity in the basal ganglia differentiates rewards from nonrewards: Temporospatial principal components analysis and source localization of the feedback negativity: Commentary. Human Brain Mapping.

[CR15] Derks B, Scheepers D, Ellemers N (2013). *The neuroscience of prejudice*.

[CR16] Doi H, Shinohara K (2012). Event-related potentials elicited in mothers by their own and unfamiliar infants’ faces with crying and smiling expression. Neuropsychologia.

[CR17] Eimer M, Holmes A (2007). Event-related brain potential correlates of emotional face processing. Neuropsychologia.

[CR18] Ellis DA, Templin TN, Naar-King S, Frey MA (2008). Toward conceptual clarity in a critical parenting construct: Parental monitoring in youth with chronic illness. Journal of Pediatric Psychology.

[CR19] Faul, F., Erdfelder, E., Buchner, A., & Lang, A.-G. (2013). G*Power (Version 3.1.7) [Computer software]. Uiversität Kiel, Germany. Retrieved from www.psycho.uni-duesseldorf.de/abteilungen/aap/gpower3/download-and-register

[CR20] Feldman R (2015). The adaptive human parental brain: Implications for children’s social development. Trends in Neurosciences.

[CR21] George C, Solomon J, Cassidy J, Shaver PR (2008). The caregiving system: A behavioral systems approach to parenting. *Handbook of attachment: Theory, research, and clinical applications*.

[CR22] Glocker ML, Langleben DD, Ruparel K, Loughead JW, Valdez JN, Griffin MD (2009). Baby schema modulates the brain reward system in nulliparous women. Proceedings of the National Academy of Sciences.

[CR23] Grasso DJ, Moser JS, Dozier M, Simons R (2009). ERP correlates of attention allocation in mothers processing faces of their children. Biological Psychology.

[CR24] Gratton G, Coles MG, Donchin E (1983). A new method for off-line removal of ocular artifact. Electroencephalography and Clinical Neurophysiology.

[CR25] Groh AM, Roisman GI, van IJzendoorn MH, Bakermans-Kranenburg MJ, Fearon R (2012). The significance of insecure and disorganized attachment for children’s internalizing symptoms: A meta-analytic study. Child Development.

[CR26] Hahn AC, DeBruine LM, Jones BC (2015). Reported maternal tendencies predict the reward value of infant facial cuteness, but not cuteness detection. Biology Letters.

[CR27] Hahn AC, Perrett DI (2014). Neural and behavioral responses to attractiveness in adult and infant faces. Neuroscience & Biobehavioral Reviews.

[CR28] Hahn AC, Symons LA, Kredel T, Hanson K, Hodgson L, Schiavone L, Jantzen KJ (2016). Early and late event-related potentials are modulated by infant and adult faces of high and low attractiveness. Social Neuroscience.

[CR29] Hallers-Haalboom ET, Groeneveld MG, van Berkel SR, Endendijk JJ, van der Pol LD, Linting M (2017). Mothers’ and fathers’ sensitivity with their two children: A longitudinal study from infancy to early childhood. Developmental Psychology.

[CR30] Hallers-Haalboom ET, Mesman J, Groeneveld MG, Endendijk JJ, van Berkel SR, van der Pol LD, Bakermans-Kranenburg MJ (2014). Mothers, fathers, sons and daughters: Parental sensitivity in families with two children. Journal of Family Psychology.

[CR31] Hofer, M. K., Buckels, E. E., White, C. J., Beall, A. T., & Schaller, M. (2017). Individual differences in activation of the parental care motivational system: An empirical distinction between protection and nurturance. *Social Psychological and Personality Science*. Advance online publication. doi:10.1177/1948550617728994 10.1037/pspp0000023 25559194

[CR32] Ispa JM, Fine MA, Halgunseth LC, Harper S, Robinson J, Boyce L (2004). Maternal intrusiveness, maternal warmth, and mother–toddler relationship outcomes: Variations across low-income ethnic and acculturation groups. Child Development.

[CR33] Klem, G. H., Lüders, H. O., Jasper, H. H., & Elger, C. (1999). The ten-twenty electrode system of the International Federation. *Electroencephalography and Clinical Neurophysiology*, *52*(Suppl. 3), 3–6. 10590970

[CR34] Langlois JH, Ritter JM, Casey RJ, Sawin DB (1995). Infant attractiveness predicts maternal behaviors and attitudes. Developmental Psychology.

[CR35] Lorenz K (1943). Die angeborenen formen möglicher erfahrung. Ethology.

[CR36] Luo LZ, Li H, Lee K (2011). Are children’s faces really more appealing than those of adults? Testing the baby schema hypothesis beyond infancy. Journal of Experimental Child Psychology.

[CR37] Malak SM, Crowley MJ, Mayes LC, Rutherford HJ (2015). Maternal anxiety and neural responses to infant faces. Journal of Affective Disorders.

[CR38] Maupin AN, Hayes NJ, Mayes LC, Rutherford HJ (2015). The application of electroencephalography to investigate the neural bases of parenting: A review. Parenting: Science and Practice.

[CR39] McElwain NL, Booth-LaForce C (2006). Maternal sensitivity to infant distress and nondistress as predictors of infant–mother attachment security. Journal of Family Psychology.

[CR40] Noll LK, Mayes LC, Rutherford HJ (2012). Investigating the impact of parental status and depression symptoms on the early perceptual coding of infant faces: An event-related potential study. Social Neuroscience.

[CR41] Papousek H, Papousek M, Bornstein MH (2002). Intuitive parenting. *Handbook of parenting*.

[CR42] Parke RD (2017). Family psychology: Past and future reflections on the field. Journal of Family Psychology.

[CR43] Peltola MJ, Yrttiaho S, Puura K, Proverbio AM, Mononen N, Lehtimäki T, Leppänen JM (2014). Motherhood and oxytocin receptor genetic variation are associated with selective changes in electrocortical responses to infant facial expressions. Emotion.

[CR44] Proverbio AM, Brignone V, Matarazzo S, Del Zotto M, Zani A (2006). Gender and parental status affect the visual cortical response to infant facial expression. Neuropsychologia.

[CR45] Proverbio AM, Riva F, Martin E, Zani A (2010). Face coding is bilateral in the female brain. PLoS ONE.

[CR46] Proverbio AM, Riva F, Zani A, Martin E (2011). Is it a baby? Perceived age affects brain processing of faces differently in women and men. Journal of Cognitive Neuroscience.

[CR47] Pryce CR, Pryce CR, Martin RD, Skuse D (1995). Determinants of motherhood in human and nonhuman primates: A biosocial model. *Motherhood in human and nonhuman primates*.

[CR48] Schneider W, Eschman A, Zuccolotto A (2002). *E-Prime user’s guide*.

[CR51] Van Der Bruggen CO, Stams GJJ, Bögels SM (2008). The relation between child and parent anxiety and parental control: A meta-analytic review. Journal of Child Psychology and Psychiatry.

[CR52] Werheid K, Schacht A, Sommer W (2007). Facial attractiveness modulates early and late event-related brain potentials. Biological Psychology.

[CR53] Young KS, Parsons CE, Stein A, Vuust P, Craske MG, Kringelbach ML (2017). The neural basis of responsive caregiving behaviour: Investigating temporal dynamics within the parental brain. Behavioural Brain Research.

[CR54] Zhang Z, Deng Z (2012). Gender, facial attractiveness, and early and late event-related potential components. Journal of Integrative Neuroscience.

